# Leaf expansion of soybean subjected to high and low atmospheric vapour pressure deficits

**DOI:** 10.1093/jxb/eru520

**Published:** 2015-01-24

**Authors:** M. Jyostna Devi, Earl W. Taliercio, Thomas R. Sinclair

**Affiliations:** ^1^Department of Crop Science, North Carolina State University, Raleigh, NC 27695, USA; ^2^Soybean and Nitrogen Fixation Unit, USDA-ARS, Raleigh, NC 27695, USA

**Keywords:** Expansin, extensin, leaf expansion, soybean, transpiration, vapour pressure deficit.

## Abstract

Vapour pressure deficit (VPD) is considered an important environmental factor that might affect leaf expansion and transpiration rate (TR) in plants. Two slow-wilting soybean (*Glycine max* (L.) Merr.) genotypes PI 416937 and PI 471938 along with commercial cultivar Hutcheson were subjected to low (1.2–1.6 kPa) and high VPD (2.8–3 kPa) environments to study their leaf expansion and TR over five days. Among the three genotypes, PI 416937 had the lowest increase in its TR (34%) at high VPD compared with low VPD and the greatest decrease in leaf area (31%). In contrast, Hutcheson had the highest increase in TR (87%) under high VPD and the lowest decrease in leaf expansion rate (18%). Expansin and extensin genes were isolated in PI 416937 to determine if changes in leaf expansion were associated with changes at the molecular level. The four studied genes were all suppressed after five days in the high VPD environment.

## Summary

Genotypic differences were found in decrease of leaf expansion with exposure to high vapour pressure deficit. Changes in leaf expansion were associated with down-regulation of expansin and extensin genes.

## Introduction

Leaf area is considered a major factor in determining crop carbon accumulation and nitrogen storage capacity, ultimately affecting crop yield. Leaf expansion leading to leaf area development is well recognized as being sensitive to soil water deficits (Tardieu and Tuberosa, 2010; Welcker *et al.*, 2007). Less well recognized is that high atmospheric vapour pressure deficit (VPD) may negatively affect leaf expansion rate, even in well-irrigated plants (Sadok *et al.*, 2007). Ehlert *et al.* (2009) found that leaf elongation rate of maize (*Zea mays* L.) was greatly influenced by high evaporative demand.

The role of water relations in controlling leaf expansion under water-deficit stress has been controversial. Some studies emphasized that leaf expansion is strongly driven by cell turgor (Bouchabké *et al.*, 2006; Hsiao *et al.*, 1998), whereas others have emphasized the role of signals from roots (Gowing *et al.*, 1990; Saab and Sharp, 1989). In the study of Ehlert *et al.* (2009) with maize, leaf expansion was highly sensitive to the VPD environment around the plants. Pressurization of the root system to overcome hydraulic conductance limitations under high VPD restored the original leaf elongation rate. The results of Munns *et al.* (2000) with barley (*Hordeum vulgare* L.) also pointed to a hydraulic component in leaf expansion as the negative effect on leaf expansion when subjected to VPD of 2 kPa disappeared when plants were kept turgid by pressurizing their root system. In addition, Ehlert *et al.* (2009) showed that treatment of roots with aquaporin inhibitors also resulted in decreased leaf elongation rate.

In soybean (*Glycine max* (L.) Merr.), transpiration rate (TR) response to changes in VPD has been shown to vary among genotypes (Fletcher *et al.*, 2007). Genotype PI 416937 expressed limited TR at high VPD (>2.2 kPa) and the response was associated with low leaf hydraulic conductance (Sinclair *et al.*, 2008). Low leaf hydraulic conductance in PI 416937 seems to be linked to aquaporins based on transpiration responses to feeding leaves aquaporin inhibitors and on gene expression studies (Sadok and Sinclair, 2010; MJ Devi *et al*., unpublished results). Even though PI 416937 has become an important parent in soybean breeding programmes (Devi *et al.*, 2014), there are no reports of the influence of VPD on leaf expansion in PI 416937.

In a recent transcriptome profiling study of PI 416937 to high VPD for four hours, most of the genes related to cell wall development such as expansins and extensins were down-regulated along with aquaporin down-regulation (MJ Devi *et al*., unpublished results). Expansins are proteins involved in cell wall loosening (Li *et al.*, 2003) and expressed in all expanding parts of the plants or in organs that undergo cell wall modifications (Gookin *et al.*, 2003; Vogler *et al.*, 2003). Up- and down-expression of expansin genes have been shown to have effects on leaf expansion, root development, and internodal development (Cho and Cosgrove, 2002; Choi *et al.*, 2003; Lee *et al.*, 2003). Extensins constitute one of the classes of the plant structural proteins that play a central role in plant form and development (Showalter, 1993). The deposition of the extensin network is important during development for correct primary wall assembly (De Tullio *et al.*, 1999), the definition of cell morphology (Cassab, 1998), and the regulation of cell elongation (Brownleader *et al.*, 2000).

The objective of this paper was to document changes in leaf expansion when exposed to differing VPD for PI 416937, a limited-TR genotype, and two genotypes (PI 471938 and Hutcheson) that exhibit no transpiration limitation under high VPD. The response of leaf expansion to low and high VPD was measured on intact plants grown in the stable environment of growth chambers. Daily TR was also determined to confirm differences among genotypes in response to VPD. Along with leaf expansion and TR, the expression of three soybean expansin and one extensin genes were measured in leaves sampled from plants exposed to prolonged high and low VPD conditions.

## Materials and methods

### Plants and growth conditions

Three soybean genotypes, PI 416937, PI 471938, and Hutcheson, were selected for this study to document the effect of VPD on development and expansion of leaves. Seeds of the three genotypes were obtained from Tommy Carter, USDA-ARS, Raleigh, NC. Two experiments were performed in the periods of 15–21 January 2013 and 12–18 February 2013. Both experiments were done in growth chambers in the Phytotron of North Carolina State University, Raleigh, NC.

Pots (20-cm diameter, 5kg soil) were filled with Gardenplus top soil (#92432, Lowes Inc., NorthWilkesboro, NC), containing 14-6.1-10 N-P-K fertilizer. Ten pots of each genotype were sown with three seeds per pot. The seeds were treated with *Bradyrhizobium* bacteria (Nitragin, Inc., Brookﬁeld, WI) before sowing. The plants were initially grown in a greenhouse for about 25 days with air temperature regulated at 30 °C day/26 °C night.

To initiate the experiment on the response to VPD, all plants were moved to a low-VPD walk-in growth chamber. The range of daytime temperature in the chamber was between 28–30 °C and the night-time temperature was 26 °C. Relative humidity in the low-VPD chamber was regulated between 66–72% by using a humidifier (Vicks ultra-sonic humidifier, V5100N). Therefore, the VPD in the chamber was 1.2–1.6 kPa.

In the evening before the start of the experiment, all pots were saturated with water and allowed to drain overnight so the plants were in a well-watered condition. The following morning after the pots were no longer dripping, the soil was covered with aluminium foil and sealed around the plant stem to avoid evaporation from the soil. The initial weight of the pots was recorded immediately after installing the aluminium foil. On all days, pots were weighed in the afternoon and differences in weight between consecutive days were used to calculate daily TR.

On each of the first two days of the experiment, all plants were kept in the low VPD chamber to measure TR and leaf area for use in subsequent normalization of results. On the third day, half the plants were moved to a high-VPD chamber resulting in five replicate plants in each VPD treatment per genotype. The daytime temperature of the high-VPD chamber was regulated between 32–35 °C and the night-time temperature was 26 °C. The relative humidity in the high-VPD chamber was at 38–42% by use of a dehumidifier (Fantech, model GD555). Consequently, the VPD in this chamber was 2.8–3.2 kPa.

The plants were kept in their respective VPD treatments for five days. Each pot was weighed daily to obtain TR. Pots were re-watered each day with the amount of lost water to avoid any water-deficit stress.

Plant leaf area expansion was estimated by calculating the difference in leaf area between successive days. Leaf area was determined for each expanding leaf on each plant based on measurement of the length of the central leaflet of each leaf and an allometric relationship between leaf area and leaflet length. Before starting the experiments, an allometric relationship for each genotype was developed by regressing trifoliolate leaf area (cm^2^) against central leaflet length (cm) (Serraj *et al.*, 1999) as follows:

$$\begin{array}{l} \text{PI 416937}:\text{ trifoliolate area}=\text{1}.\text{292}\times {\left[\text{central leaflet length}\right]}^{\text{2}}\\ \;\left({\text{R}}^{2}=0.\text{98}\right) \end{array}$$

$$\begin{array}{l} \text{PI 471938}:\text{ trifoliolate area}=\text{1}.\text{254}\times {\left[\text{central leaflet length}\right]}^{\text{2}}\\ \;\left({\text{R}}^{2}=0.\text{97}\right) \end{array}$$

$$\begin{array}{l} \text{Hutcheson}:\text{ trifoliolate area}=\text{1}.\text{38}0\times {\left[\text{central leaflet length}\right]}^{\text{2}}\\ \;\left({\text{R}}^{2}=0.\text{97}\right) \end{array}$$

Total leaf area per plant was obtained by summing the area of the individual trifoliolate leaves.

Total number of leaflets per plant in two treatments was counted before the start of the experiment and at the end of the experiment. Number of leaflets produced during the experimental period was calculated as the difference between the measured values at the beginning and end of the experiment.

### RNA isolation and real time quantitative PCR

Three replicate leaf samples for RNA isolation were collected in the second experiment from both the low and high VPD treatments on the fifth day after the VPD treatments were initiated. The samples were stored at –80 °C until they could be processed for molecular analysis. Total RNA was extracted from the leaf using the Qiagen RNeasy plant kit according to the manufacturer’s instructions. After DNaseI treatment (Ambion), RNA was quantified by measurement on a NanoDrop 1000 (Thermo Fisher, Waltham, MA), and the integrity and concentration was confirmed by running the samples on a denaturing agarose gel.

The selection of the expansin and extensin genes to be evaluated was aided by a transcriptome profiling experiment determining the effects of high VPD on gene expression of leaves from PI 416937 (MJ Devi *et al*., unpublished results). Nine expansin genes and four extensin genes were down-regulated when exposed to high VPD (data not shown). However, the effect of high VPD on leaf expansion was not measured. Based on the largest changes in gene expression with exposure to high VPD, three expansin genes (Glyma01g06030, Glyma11g26240, and Glyma20g04490) and one extensin gene (Glyma13g24720) were selected for determination of expression in the current study.

Genes EF-1 (elongation factor-1) and UKN-2 (unknown function-2) of soybean were used to normalize all values in the QRT-PCR assays. Expression of these genes have been reported to be stable across developmental stages and under abiotic stress in soybean (Hu *et al.*, 2009), and the stability of expression of these genes was consistent with the observations in the treatments used in these experiments. Primers for QRT-PCR were designed using Primer3 software. Primer sequences are listed in Table 1. QRT-PCR reactions were performed using SYBR Green iTaq Universal probes one-step kit, 500×20 µl reactions (# 172–5141, Bio-rad) on a Bio-rad CFX Real-Time PCR Detection System (Bio-Rad, Hercules, CA, USA) following the manufacturer’s instructions. One microliter of synthesized cDNA (diluted 1:10) was used as template. Primer efficiency was determined as explained in Pfaffl (2001). The amplification reactions consisted of a 2-min denaturing step at 95 °C, followed by 40 cycles at 95 °C for 10 s, 60 °C for 30 s, and 70 °C for 30 s, ending with a melting curve program at 70 °C for 30 s. Three replicate reactions per sample were used to ensure statistical significance. The RNA from each sample was analysed simultaneously. Expression levels for all candidate genes were computed based on the stable expression level of the reference gene as described by Pfaffl (2001). Expansin and extensin gene expression under high VPD were calculated relative to the gene expression under low VPD. Analyses of the melting curve were consistent with a single amplicon.

**Table 1.** Phytozome accession number, its tentative annotation, primer efficiency, forward and reverse primers, and Tm of the primer sequences used in the study

Gene	Tentative annotation	Primer efficiency	Forward sequence	Reverse sequence	Tm (°C)
Glyma01g06030.1	Expansin	1.85	CTCAATTCCGCTAGAGAGGC	CATACACTAACACTTAAATTGAGG	52
Glyma20g04490.1	Expansin	1.89	TGTTGTCCAAAGTTCTAAGTCAC	CTAGTATACTAACCAGTCATCA	52
Glyma11g26240.1	Expansin	1.86	ACATTATTGCAAATTTACAATGGG	GAATCAATACCTATTAATGACAC	52
Glyma13g24720.1	Extensin	2.00	CGCAGTTCAAGGAGTTGTTTATG	TCACAACGGCACCAAGTAG	52
Ef-1		2.00	CTG TAA CAA GAT GGA TGC CAC TAC	CAG TCA AGG TTT GTG GAC CT	55.5
UKN 2		1.90	GCC TCT GGA TAC CTG CTC AAG	ACC TCC TCC TCA AAC TCC TCT G	57.4

### Data analysis

The effects of VPD on TR and leaf area expansion rate were compared among genotypes by normalization so the initial reference data were all centred on a value of one. The normalization was done using the data measured under low VPD conditions for two days before the start of the experiment. The daily TR and leaf expansion rate during the treatment period following the initial two days was divided by the average TR and leaf area expansion rate for the initial two days.

Leaf expansion rate per day, leaflet number, leaf area, and gene expression data were analysed for their genotypic differences using ANOVA by Tukey’s method.

## Results

### Transpiration rate

Daily TR was measured for the three genotypes over five days of exposure to differing VPD environments. The TR results between the two experiments for each VPD treatment and genotype were not significantly different (*P*<0.05), so the data for the two experiments were pooled. The TR differences between the high and low VPD treatments measured on the first day remained the same over all days of the experiments. As expected, the normalized transpiration was greater in the high VPD as compared with the low VPD treatment for all three genotypes. However, the amount of transpiration increase differed substantially among genotypes. The average increase over all days in TR for the high VPD treatment was 34.5±2.79% for slow-wilting VPD-sensitive PI 416937, 58.5±5.87% for slow-wilting VPD-insensitive PI 471938, and 86.8±4.97% for fast-wilting VPD-insensitive Hutcheson.

### Leaf expansion

**Fig. 1.**
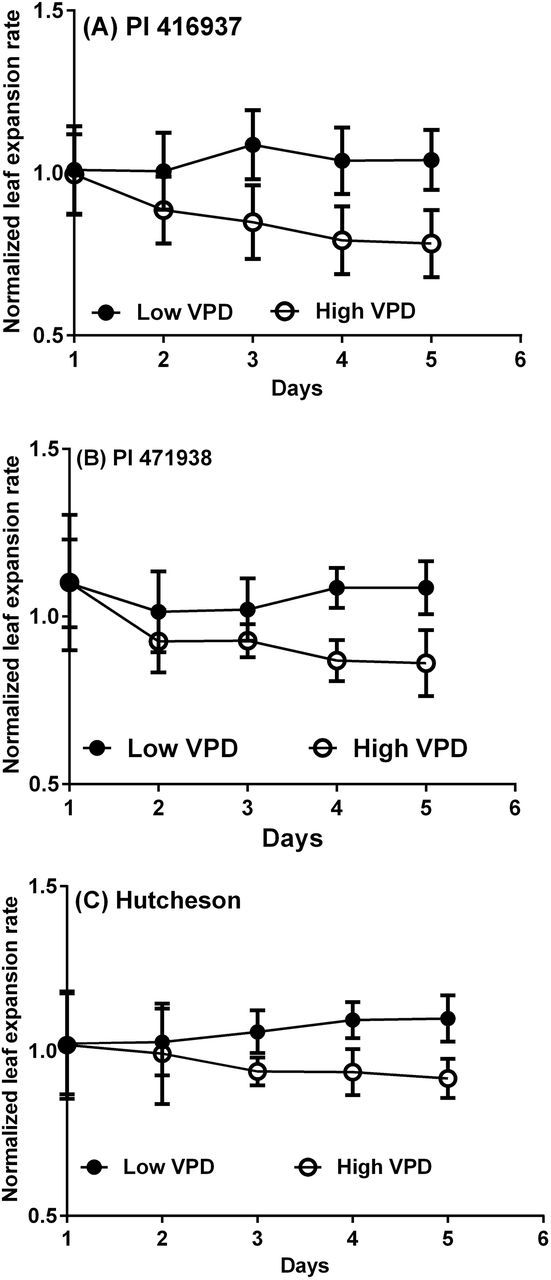
Mean normalized leaf expansion rate of (A) PI 416937 (B) PI 471938 and (C) Hutcheson during the VPD treatments following the initial two days of measurement in the low VPD chamber. The results were plotted against days since the initiation of the treatments in the high VPD chamber (open circles) and in the low VPD chamber (closed circles). The standard error bars are plotted for the mean on each day.

Similarly to TR, leaf expansion rate results between the two experiments did not differ significantly (*P*<0.05) for each VPD treatment and genotype. Again, the data for the two experiments were pooled. These results showed that the temporal changes in leaf area expansion in response to VPD were different from the TR response (Fig. 1). On the first day following imposition of the two different VPD treatments, there was no change in leaf expansion rate. On Day 2, the rates were not significantly different between the two VPD treatments, though a trend seemed to be emerging leading to differences. On the third day for PI 416937 and fourth day for PI 471938 and Hutcheson, the leaf expansion rate in the high VPD treatment was significantly less than the low VPD treatment. The differences between the two VPD treatments for all three genotypes were essentially stable on Day 4 and 5. The average decrease in leaf expansion during the last two days was significantly different among genotypes with values of decrease of 31.9%±0.99 for PI 416937, 25.5±0.57 for PI 471938, and 18.1%±2.11 for Hutcheson.

###  Leaflet number

**Fig. 2.**
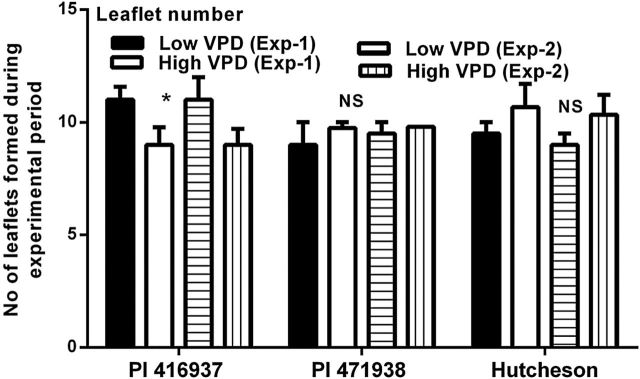
Mean of leaflets formed during experimental period for each VPD treatment and each experiment by PI 416937, PI 471938, and Hutcheson. The standard error bars are plotted for each treatment. The asterisk (*) shows values are significantly different at *P*<0.05 based on *t*-test, and NS indicates non-significance.

The number of leaflets formed during the experimental period was significantly different (*P*<0.05) from low to high VPD in PI 416937 in both the experiments, but did not vary in PI 471938 and Hutcheson (Fig. 2). For PI 416937, the number of leaflets formed in low VPD conditions was 11±0.57 and 11±1.0 in Experiment 1 and 2, respectively, whereas in high VPD conditions the leaflet number was 9±0.74 and 9±0.70 in Experiment 1 and 2, respectively.

### Expansin and extensin gene expression

**Fig. 3.**
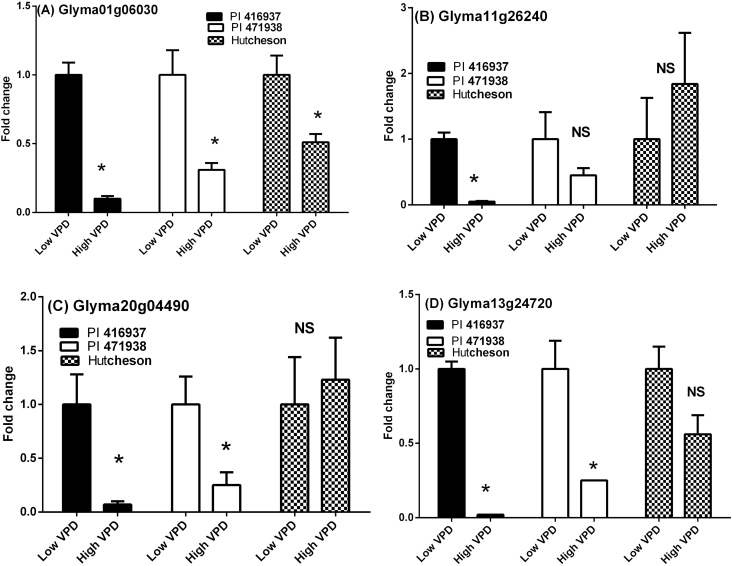
Relative expression of three expansin genes (A) Glyma01g06030, (B) Glyma11g26240, (C) Glyma20g04490, and one extensin gene (D) Glyma13g24720 in PI 416937, PI 471938, and Hutcheson under high VPD conditions. The standard error bars are plotted for each treatment. The asterisk (*) shows significant difference between VPD treatments at *P*<0.05 and NS indicates non-significance.

The three expansin genes and one extensin gene were generally down-regulated under the high VPD treatment as compared with the low VPD treatment, consistent with transcriptome profiling data (MJ Devi *et al*., unpublished results), although there were some genotype-specific effects (Fig. 3). The difference in expression was especially marked for PI 416937 in which the expression of all genes under high VPD was near zero. Expression of the expansin Glyma01g06030 gene (Fig. 3A) for all genotypes was down-regulated in the high VPD treatment as compared with the low VPD treatment. The high VPD treatment resulted in down-regulation of expansin Glyma11g26240 in PI 416937 but there was no significant difference between the VPD treatments for the other two genotypes (Fig. 3B). For expansin gene Glyma20g04490 and extensin gene Glyma13g24720, there was down-regulation at high VPD for PI 416937 and PI 471938 but there was no significant difference in Hutcheson (Fig. 3C, D).

## Discussion

An objective of this study was to document the influence of VPD on leaf expansion rate of three soybean genotypes, which exhibit differences in their TR responses to increasing VPD. The results presented here showed that all three genotypes exhibited depressed leaf area development under elevated VPD (Fig. 1). The decrease in leaf area under the high VPD treatment (Fig. 2) was associated with a decreased leaf expansion but not with a reduction in leaflet number as the number was only slightly decreased (PI 416937) or not at all (PI 471938 and Hutcheson). The observed sensitivity of leaf expansion in these soybean plants to elevated VPD is consistent with observations of leaf expansion in miscanthus and maize (Bouchabké *et al.*, 2006; Clifton-Brown and Jones, 1999), even though previous studies were performed on individual leaves by exposing them to short term (minutes to hours) VPD changes. Furthermore, in the studies of Clifton-Brown and Jones (1999) and Bouchabké *et al*. (2006), the decrease in leaf expansion rate in response to high VPD was transient. In the current study, the full effect of the high VPD treatment developed over several days resulting in lower expansion rates.

The genotypes differed in their decrease in leaf expansion under high VPD conditions. PI 416937 had the largest decrease followed by PI 471938, and Hutcheson was the least sensitive. The decrease in leaf expansion of PI 416937 was almost double that of Hutcheson. This difference among genotypes was the opposite of the transpiration response to high VPD. The TR increase under high VPD for PI 416937 was only 34%, whereas the increase by PI 471938 was 58% and by Hutcheson was 87%.

The difference in long-term water loss among genotypes is consistent with the reported differences in expression of the limited-TR trait in these genotypes. Limited TR has been reported in PI 416937 at VPD greater than about 2.2 kPa (Fletcher *et al.*, 2007; Sinclair *et al.*, 2008), whereas no limitation on TR at high VPD was reported for PI 471938 (Fletcher *et al.*, 2007) or Hutcheson when measured in temperatures of the current experiment (Seversike *et al.*, 2013). As the basis for the limited-TR in PI 416937 was linked to a low hydraulic conductance in the xylem-to-guard-cell pathway in the leaves of this genotype (Sinclair *et al.*, 2008), it seems a different mechanism is needed to explain the decrease in leaf expansion under high VPD. A possibility explored in this study was that expression of expansin and extensin genes might be altered under high VPD.

Expansin and extensin genes are involved in cell wall loosening, growth maintenance, and development. When plants are exposed to water-deficit conditions, cell walls of the leaf tissues becomes less extensible reducing tissue expansion rate (Cosgrove, 2005). Several expansin genes were affected by drought stress and their expression was well correlated with the elongation rate in leaves (Muller *et al.*, 2007; Wu and Cosgrove, 2000). Down-regulation of several cell wall extensins were found in a leaf-elongation tolerant variety of rice (*Oryza sativa* L.) and up-regulated in a sensitive variety (Cal *et al.*, 2013). Suppression of most expansin genes in *Arabidopsis* resulted in repression of growth during the later stage of leaf development (Goh *et al.*, 2012).

In tests with PI 416937 (MJ Devi *et al*., unpublished results), after 4h exposure to high VPD three expansin genes and one extensin gene were identified showing substantial down-regulation. These results were confirmed in this study using leaves harvested from plants subject to five days of VPD treatment (Fig. 3). The lowest expression of all four genes was observed in PI 416937 in comparing the high VPD treatment with the low VPD treatment. Down-regulation was also observed for all four genes in PI 471938 but not nearly to the same extent as in PI 416937. This quantitative difference aligns with the greater loss in leaf expansion in PI 416937 as compared with PI 471938 under high VPD.

The transcript number of Hutcheson for these four genes was mixed. Two genes, expansin Glyma01g06030 and extensin Glyma13g24720, showed decreases, although not to the level of either of the other genotypes. There was no change in transcript number for the other two genes that were studied. The smaller change or no change in transcript number is consistent with the comparatively small change in leaf expansion under high VPD.

Overall, genotypic differences in leaf area development under high VPD were clearly demonstrated. Although little or no change in leaf appearance was shown in this study, differences were observed in overall plant leaf area under high VPD. Therefore, these results showed that leaf area expansion is a key difference among these soybean lines to high VPD. The comparison among genotypes showed a negative relationship between changes in TR and leaf area expansion under high VPD. That is, under high VPD, higher TR was associated with lower leaf area expansion rates as shown in the results for Hutcheson. The results of PI 416937 are particularly interesting because expression of the limited TR and lower plant water loss was associated with the greatest loss in leaf expansion. The loss in leaf expansion was associated with decreased number of transcripts for the four genes related to leaf expansion that were documented in this study. These results indicate the possibility of an adjustment in transcript number in response to changes in the VPD environment. The linkage between VPD environment and gene expression is unknown but seems to offer a fertile opportunity for future research.

## Abbreviations:

TR: transpiration rate
VPD: vapour pressure deficit.

## References

[CIT0001] Bouchabké O Tardieu F Simonneau T 2006 Leaf growth and turgor in growing cells of maize (*Zea mays* L.) respond to evaporative demand under moderate irrigation but not in water-saturated soil. Plant, Cell and Environment 29, 1138–1148. 10.1111/j.1365-3040.2005.01494.x 17080939

[CIT0002] Brownleader M D Hopkins J Mobasheri A Dey P M Jackson P Trevan M 2000 Role of extensin peroxidase in tomato (*Lycopersicon esculentum* Mill.) seedling growth. Planta 210, 668–676. 10787062 10.1007/s004250050058

[CIT0003] Cal AJ Liu D Mauleon R Hsing Y-I C Serraj R 2013 Transcriptome profiling of leaf elongation zone under drought in contrasting rice cultivars. PLoS One 8, e54537. 23372737 10.1371/journal.pone.0054537 PMC3553057

[CIT0004] Cassab GI 1998 Plant cell wall proteins. Annual Review of Plant Physiology and Plant Molecular Biology 49, 281–309. 10.1146/annurev.arplant.49.1.281 15012236

[CIT0005] Cho H-T Cosgrove DJ 2002 Regulation of root hair initiation and expansin gene expression in *Arabidopsis* . The Plant Cell Online 14, 3237–3253. 10.1105/tpc.006437 PMC151215 12468740

[CIT0006] Choi D Lee Y Cho H-T Kende H 2003 Regulation of expansin gene expression affects growth and development in transgenic rice plants. The Plant Cell Online 15, 1386–1398. 10.1105/tpc.011965 PMC156374 12782731

[CIT0007] Clifton-Brown JC Jones MB 1999 Alteration of TR, by changing air vapour pressure deficit, influences leaf extension rate transiently in *Miscanthus* . Journal of Experimental Botany 50, 1393–1401.

[CIT0008] Cosgrove DJ 2005 Growth of the plant cell wall. Nature Reviews Molecular Cell Biology 6, 850–861. 16261190 10.1038/nrm1746

[CIT0009] De Tullio MC Paciolla C Dalla Vecchia F Rascio N D’Emerico S De Gara L Liso R Arrigoni O 1999 Changes in onion root development induced by the inhibition of peptidyl-prolyl hydroxylase and influence of the ascorbate system on cell division and elongation. Planta 209, 424–434. 10550623 10.1007/s004250050745

[CIT0010] Devi MJ Sinclair TR Chen P Carter T 2014 Evaluation of elite southern maturity soybean breeding lines for drought tolerant traits. Agronomy Journal 106, 1947–1954.

[CIT0011] Ehlert C Maurel C Tardieu FO Simonneau T 2009 Aquaporin-mediated reduction in maize root hydraulic conductivity impacts cell turgor and leaf elongation even without changing transpiration. Plant Physiology 150, 1093–1104. 19369594 10.1104/pp.108.131458 PMC2689965

[CIT0012] Fletcher AL Sinclair TR Allen Jr LH 2007 Transpiration responses to vapor pressure deficit in well watered slow wilting and commercial soybean. Environmental and Experimental Botany 61, 145–151.

[CIT0013] Goh HH Sloan J Dorca-Fornell C Fleming A 2012 Inducible repression of multiple expansin genes leads to growth suppression during leaf development. Plant Physiology 159, 1759–1770. 22740614 10.1104/pp.112.200881 PMC3425211

[CIT0014] Gookin TE Hunter DA Reid MS 2003 Temporal analysis of alpha and beta-expansin expression during floral opening and senescence. Plant Science 164, 769–781.

[CIT0015] Gowing DJG Davies WJ Jones HG 1990 A positive root-sourced signal as an indicator of soil drying in apple, *Malus × domestica* Borkh. Journal of Experimental Botany 41, 1535–1540.

[CIT0016] Hsiao TC Frensch J Rojas-Lara BA 1998 The pressure-jump technique shows maize leaf growth to be enhanced by increases in turgor only when water status is not too high. Plant, Cell and Environment 21, 33–42.

[CIT0017] Hu R Fan C Li H Zhang Q Fu Y-F 2009 Evaluation of putative reference genes for gene expression normalization in soybean by quantitative real-time RT-PCR. BMC Molecular Biology 10, 93. 19785741 10.1186/1471-2199-10-93 PMC2761916

[CIT0018] Lee D-K Ahn JH Song S-K Choi YD Lee JS 2003 Expression of an expansin gene is correlated with root elongation in soybean. Plant Physiology 131, 985–997. 12644651 10.1104/pp.009902 PMC166864

[CIT0019] Li Y Jones L McQueen-Mason S 2003 Expansins and cell growth. Current Opinion in Plant Biology 6, 603–610. 14611960 10.1016/j.pbi.2003.09.003

[CIT0020] Muller B Bourdais G Reidy B Bencivenni C Massonneau A Condamine P Rolland G Conéjéro G Rogowsky P Tardieu F 2007 Association of specific expansins with growth in maize leaves is maintained under environmental, genetic, and developmental sources of variation. Plant Physiology 143, 278–290. 17098857 10.1104/pp.106.087494 PMC1761972

[CIT0021] Munns R Passioura JB Guo J Chazen O Cramer GR 2000 Water relations and leaf expansion: importance of time scale. Journal of Experimental Botany 51, 1495–1504. 11006301 10.1093/jexbot/51.350.1495

[CIT0022] Pfaffl MW 2001 A new mathematical model for relative quantification in real-time RT-PCR. Nucleic Acids Research 29, e45. 11328886 10.1093/nar/29.9.e45 PMC55695

[CIT0023] Saab IN Sharp RE 1989 Non-hydraulic signals from maize roots in drying soil: inhibition of leaf elongation but not stomatal conductance. Planta 179, 466–474. 24201770 10.1007/BF00397586

[CIT0024] Sadok W Naudin P Boussuge B Muller B Welcker C Tardieu F 2007 Leaf growth rate per unit thermal time follows QTL-dependent daily patterns in hundreds of maize lines under naturally fluctuating conditions. Plant, Cell and Environment 30, 135–146. 10.1111/j.1365-3040.2006.01611.x 17238905

[CIT0025] Sadok W Sinclair TR 2010 Transpiration response of slow wilting and commercial soybean (*Glycine max* (L.) Merr.) genotypes to three aquaporin inhibitors. Journal of Experimental Botany 61, 821–829. 19969533 10.1093/jxb/erp350 PMC2814113

[CIT0026] Serraj R Allen LH Sinclair TR 1999 Soybean leaf growth and gas exchange response to drought under carbon dioxide enrichment. Global Change Biology 5, 283–291.

[CIT0027] Seversike TM Sermons SM Sinclair TR Carter TE Jr Rufty TW 2013 Temperature interactions with transpiration response to vapor pressure deficit among cultivated and wild soybean genotypes. Physiologia Plantarum 148, 62–73. 22989317 10.1111/j.1399-3054.2012.01693.x

[CIT0028] Showalter AM 1993 Structure and function of plant cell wall proteins. The Plant Cell 5, 9–23. 8439747 10.1105/tpc.5.1.9 PMC160246

[CIT0029] Sinclair TR Zwieniecki MA Holbrook NM 2008 Low leaf hydraulic conductance associated with drought tolerance in soybean. Physiologia Plantarum 132, 446–451. 18333998 10.1111/j.1399-3054.2007.01028.x

[CIT0030] Tardieu F Tuberosa R 2010 Dissection and modelling of abiotic stress tolerance in plants. Current Opinion in Plant Biology 13, 206–212. 20097596 10.1016/j.pbi.2009.12.012

[CIT0031] Vogler H Caderas D Mandel T Kuhlemeier C 2003 Domains of expansin gene expression define growth regions in the shoot apex of tomato. Plant Molecular Biology 53, 267–272. 14750517 10.1023/b:plan.0000006999.48516.be

[CIT0032] Welcker C Boussuge B Bencivenni C Ribaut JM Tardieu F 2007 Are source and sink strengths genetically linked in maize plants subjected to water deficit? A QTL study of the responses of leaf growth and of Anthesis-Silking Interval to water deficit. Journal of Experimental Botany 58, 339–349. 17130185 10.1093/jxb/erl227

[CIT0033] Wu Y Cosgrove DJ 2000 Adaptation of roots to low water potentials by changes in cell wall extensibility and cell wall proteins. Journal of Experimental Botany 51, 1543–1553. 11006305 10.1093/jexbot/51.350.1543

